# Finding the Intersection of Neuroplasticity, Stroke Recovery, and Learning: Scope and Contributions to Stroke Rehabilitation

**DOI:** 10.1155/2019/5232374

**Published:** 2019-05-02

**Authors:** Leeanne Carey, Alistair Walsh, Achini Adikari, Peter Goodin, Damminda Alahakoon, Daswin De Silva, Kok-Leong Ong, Michael Nilsson, Lara Boyd

**Affiliations:** ^1^Occupational Therapy, School of Allied Health, Human Sciences and Sport, College of Science, Health and Engineering, La Trobe University, Bundoora, VIC 3086, Australia; ^2^Neurorehabilitation and Recovery, Stroke Division, Florey Institute of Neuroscience and Mental Health, Heidelberg VIC 3084, Australia; ^3^Research Centre for Data Analytics and Cognition, La Trobe University, Bundoora, VIC 3086, Australia; ^4^Department of Medicine and Neurology, Melbourne Brain Centre, Royal Melbourne Hospital, Parkville, VIC 3050, Australia; ^5^Faculty of Health and Medicine and Centre for Rehab Innovations, The University of Newcastle, Callaghan NSW 2308, Australia; ^6^LKC School of Medicine, Nanyang Technological University (NTU), 308232, Singapore; ^7^Djavad Mowafaghian Centre for Brain Health, Faculty of Medicine, University of British Columbia, Vancouver, BC, Canada V6T 1Z3

## Abstract

**Aim:**

Neural plastic changes are experience and learning dependent, yet exploiting this knowledge to enhance clinical outcomes after stroke is in its infancy. Our aim was to search the available evidence for the core concepts of neuroplasticity, stroke recovery, and learning; identify links between these concepts; and identify and review the themes that best characterise the intersection of these three concepts.

**Methods:**

We developed a novel approach to identify the common research topics among the three areas: neuroplasticity, stroke recovery, and learning. A concept map was created *a priori*, and separate searches were conducted for each concept. The methodology involved three main phases: data collection and filtering, development of a clinical vocabulary, and the development of an automatic clinical text processing engine to aid the process and identify the unique and common topics. The common themes from the intersection of the three concepts were identified. These were then reviewed, with particular reference to the top 30 articles identified as intersecting these concepts.

**Results:**

The search of the three concepts separately yielded 405,636 publications. Publications were filtered to include only human studies, generating 263,751 publications related to the concepts of neuroplasticity (*n* = 6,498), stroke recovery (*n* = 79,060), and learning (*n* = 178,193). A cluster concept map (network graph) was generated from the results; indicating the concept nodes, strength of link between nodes, and the intersection between all three concepts. We identified 23 common themes (topics) and the top 30 articles that best represent the intersecting themes. A time-linked pattern emerged.

**Discussion and Conclusions:**

Our novel approach developed for this review allowed the identification of the common themes/topics that intersect the concepts of neuroplasticity, stroke recovery, and learning. These may be synthesised to advance a neuroscience-informed approach to stroke rehabilitation. We also identified gaps in available literature using this approach. These may help guide future targeted research.

## 1. Introduction

*Neuroplasticity* can be defined as the ability of the nervous system to respond to intrinsic or extrinsic stimuli by reorganizing its structure, function, and connections [1]. Neural plastic changes are associated with development [2] and learning [3, 4]. They occur throughout the lifespan [5] and may be enhanced following injury [6]. They are influenced by experience [7] and the context [8, 9] in which that experience occurs. The major drivers of neuroplastic change are meaningful behavior [10]. Evidence of neural plastic changes can be observed at various levels, e.g., cellular/synaptic changes, changes in the structure and function of brain regions and networks, and changes in behavior such as improved skill and adaptability [11, 12]. Strong scientific evidence demonstrates that the brain has remarkable capacity for plasticity and reorganisation, yet exploiting this knowledge to enhance clinical outcomes is in its infancy.

After a brain injury, such as stroke, the person is challenged to sense, move, communicate, and engage in daily activities with the brain and body that are impacted by the stroke. Immediate and long-term effects of stroke include impairment in sensation, movement, cognition, psychological and emotional functions, and reduced independence and quality of life. There may be evidence of improvement and some regaining of lost skill. A trajectory of spontaneous and supported recovery over the days, weeks, and months after stroke has been described [13, 14]. Yet rehabilitation outcomes are currently suboptimal and variable [15, 16], and evidence supporting novel or more effective treatments is limited.

Neural plastic changes occur following brain injury, such as stroke [17]. The changes may occur in the days, weeks, months, and years following stroke [11, 13]. They may be adaptive or maladaptive [18, 19]. For example, a person can learn nonuse of the limb or develop dystonic postures following sensory loss [20]. However, we have yet to harness this window of opportunity for ongoing recovery both short- and long-term after stroke. The continuum of recovery after stroke presents opportunities for targeted rehabilitation to harness and enhance these mechanisms of neural plasticity for improved outcomes.

Neural plastic changes are *experience* and *learning dependent*. *Learning* is the process of acquiring a relatively lasting change in knowledge and skills [21]. Learning cannot be measured directly, and assessment may address different criterion indicators of learning [21]. The potential exists for the phenomenon of neural plasticity to be shaped by the experiences that occur following stroke [8, 9, 19] and to be positively impacted by rehabilitation [9, 19, 22]. The question is how can we build on and shape this experience and drive positive plasticity to achieve better outcomes for stroke survivors?

*Neurorehabilitation* may be defined as “facilitation of adaptive learning” [23]. *Stroke rehabilitation* founded on neuroscience is now recognised for its capacity to achieve more restorative outcomes [1, 19]. Experience and learning-dependent plasticity are core to this change [12, 23]. There are different conditions under which that plasticity may be enhanced, facilitated, and/or consolidated. These different conditions likely impact the type of neuroplasticity facilitated and behavioral outcomes observed. An advanced understanding of these will help guide the development of neuroscience-based interventions.

The aim of our scoping review was (i) to search the evidence available in relation to the three core concepts of neural plasticity, stroke recovery, and learning; (ii) to identify how these concepts are linked to each other; and (iii) to identify and discuss the themes/topics that best characterise the intersection of these three concepts, in order to better inform the neuroscience basis of stroke rehabilitation and stroke recovery.

In relation to neural plasticity, we were interested in the identification of evidence of neuroplastic changes, e.g., at cellular and neural network levels. This included evidence such as synaptic changes, brain networks, and functional connectivity. We anticipated this literature would be primarily found in neuroscience and neuroimaging type journals. For the concept of stroke recovery, we were interested in outcomes related to impairment, performance, participation, and quality of life, at different times in the recovery trajectory and in relation to rehabilitation. The concept of *learning* focused on the process of change and included domains such as experience, different types of learning, attention and cognition, adaptation, environment, motivation, and goal. Investigation of the *links and intersection* between these concepts has the potential to reveal the following: (1) the type of learning experience that can enhance neural plasticity; (2) the evidence that links neural plasticity and improved outcomes for stroke survivors; and (3) how the different learning experiences linked with neural plasticity might influence/contribute to better stroke outcomes.

In achieving our aim, we sought to develop and use a methodology that would enable a broad and comprehensive scoping of the current literature. This included identification of key topics represented in the literature that relate to the three core concepts and an approach that permits searching and identification of related terms that may be used by authors. This was important to maximise the likelihood that a broad range of terms that are likely to have similar or overlapping meaning was able to be searched and accessed.

## 2. Methodology

**Figure 1:**
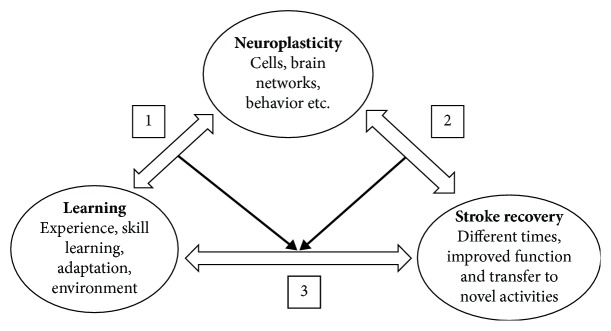
Concept map depicting the three main concepts and the potential associations between them.

A series of steps were conducted to identify the common research interests among the three research areas: neuroplasticity, stroke recovery, and learning. A concept map was first developed to guide the review in relation to our aim.  Figure 1 depicts the concept map comprising (a) the three main concepts (neuroplasticity, stroke recovery, and learning); (b) example main keywords related to each of the concepts; (c) arrows depicting the associations among each of the main concepts; and (d) numbers to indicate our key foci/associations of interest. The target population was adult humans with stroke. Health outcomes included improved function, such as skill, performance, and quality of life.

Following the initial creation of the concept map, our approach was to scope the literature available in relation to each of the three core concepts separately and then identify the relationship (link) between each other. Given the amount of literature for each of the concepts, we adopted a novel approach to searching and clustering the large number of papers and identifying the links and intersection. In particular, we employed an automatic text processing engine (Section 2.3) to aid the process and identify the unique and common topics among these research concepts. In this way, we were able to map the identified topics to components 1, 2, and 3 in the proposed concept map. A narrative review was then conducted of the common themes and the top articles that were identified as intersecting the three concepts.

Our novel approach consisted of three main phases: data collection and filtering, development of a clinical vocabulary, and the development of an automatic clinical text processing engine. The methodology to build the vocabulary and text processing engine is comprised of three main technical approaches: *text mining* [24]*—*to extract relevant information from the research articles and structure data according to our analysis; *natural language processing* (NLP) [25]—to create word embeddings and topic ontology; and *text analysis* [26]*—*to derive insights on how the three concepts are linked together based on the identified topic associations. The details of the techniques are further described in Sections 2.2 and 2.3.

### 2.1. Data Collection and Filtering

A comprehensive literature search was conducted using PubMed to assemble research studies addressing neuroplasticity, stroke recovery, and learning. First, we conducted three separate and broad searches. We used the tree of MeSH headings associated with each of these concepts to ensure broad and comprehensive inclusion of data. For example, under the heading of learning [F02.463.425], this included 25 subheadings and further 32 subheadings under these subheadings. As an inclusion criteria for the collected studies, we selected research where experiments were conducted on humans.

The PubMed database was accessed using the Entrez Programming Utilities (E-utilities), a set of eight server side programs that provide a programmatic interface to the National Center for Biotechnology Information (NCBI) database system [27]. A python helper library, used to interact with the E-utilities and perform other formatting and data managing tasks, is available at https://github.com/alistairwalsh/informatician.

The three separate requests with the query terms “neuroplasticity[MeSH],” “stroke[MeSH],” and “learning[MeSH]” returned associated PubMed ID numbers, which were then used to retrieve all the information available for those articles. The resulting XML documents were then searched for an English abstract along with their article title, abstract, and index terms (i.e., mesh terms and/or keyword lists) to produce a collection of studies that were searched for terms of interest.

Three sources of data were collected and analysed for each article retrieved: title, abstract, and index terms, as identified in the article by the authors. This data was not only selected for its availability but also based on the expectation that key topic words should be captured in these sources. Further, data collected across these data sources should be comparable as the type of information included in abstracts is relatively uniform, with clear expectations, and is usually word limited, thus minimising bias due to variance in article length.

### 2.2. Development of a Clinical Vocabulary

Following the filtering of the collected documents, text mining tasks were performed to gain insights on the associations between the three concepts. Text mining is the process of extracting useful information from unstructured data and customization according to the requirements. For this purpose, it was necessary to build a vocabulary/initial seed word list, which could be used as the guide for text mining to extract relevant information. Therefore, a clinical vocabulary comprising of prominent topics in all three research areas was required. The following steps were undertaken to develop the vocabulary.

#### 2.2.1. Domain Knowledge from Experts

An initial vocabulary was formed using the domain knowledge from experts. These topic vocabulary terms are listed in Table 1. This initial vocabulary included keywords as well as key phrases. Three knowledge experts (LC, MN, and LB) contributed to the list.

#### 2.2.2. Incorporating Index Terms Provided by Authors in Articles Retrieved

Index terms (keywords provided by authors) and MESH terms used by the authors for each article were included to further enrich the vocabulary.

#### 2.2.3. Word Embedding Technique to Expand the Vocabulary

Word embedding is a machine learning technique that intelligently captures the context of a word in a document, i.e., capturing semantic and syntactic similarity as well as identifying the relation with other words. This technique was used to extract synonyms for the original list of terms (i.e., as outlined in Table 1). The extracted model was applied to the three sources of data from each article (i.e. title, abstract, and index terms). A word2vec model was trained from the collection of publications that can identify terms that were being used in a similar context. For instance, the word “consolidation” generated a similarly used word list (“formation,” “reconsolidation,” “storage,” and “acquisition”). The generated similar words were manually reviewed for relevance before adding to the vocabulary.

### 2.3. Development of an Automatic Clinical Text Processing Engine

To analyse the associations between the concepts, we developed an automatic clinical text processing engine, which is capable of automatically extracting key terms from documents and generating a concept link map. A series of natural language processing (NLP) techniques and text analysis were used for this purpose. NLP is known as the application of computational techniques to analyse natural language which is unstructured textual data [28]. The developed text processing engine is comprised of an array of NLP techniques to extract topics, calculate similarity, and create a concept link map which was used for the analysis of topic associations. The primary tasks of the developed engine are explained below.

#### 2.3.1. Automatic Term Extraction

Intelligent search algorithms [29] were used to automatically extract relevant terms from the abstracts, titles, and index terms provided by the authors of the publications. The developed vocabulary was used for this purpose. The process generated lists of topics being discussed for each publication.

#### 2.3.2. Term Similarity Identification

Once the terms were extracted, it was essential to identify the common terms between the three groups. We used NLP techniques to automatically group publications that have similar topics and thereby identify unique and common clusters of topics.

#### 2.3.3. Weight Concept Link Map

The results were then used to generate a weighted concept link map illustrating the topics that connect the concepts together. The output concept map represented an overview of the topics that link the three concepts together. Each connection was given a score based on the number of publications, therefore allowing filtering out only the important connections.

**Figure 2:**
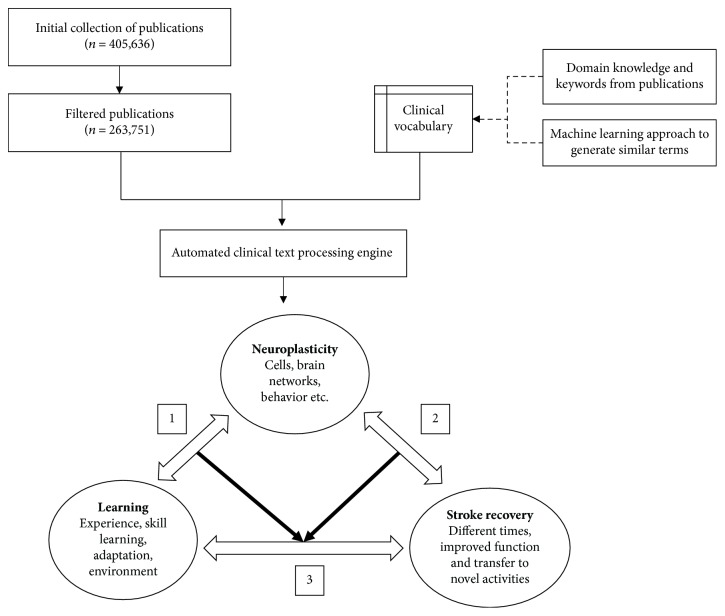
The high-level process of the methodology.

The high-level process of the text analysis engine is illustrated in Figure 2.

### 2.4. Investigation of Time-Linked Patterns in Keywords Used for Each Concept

We conducted a post hoc analysis to explore if any time-related patterns emerged in relation to the emergence of topics for each of the three concepts over time. First, the three core concepts were analysed with the date of the publication and for each topic; a percentage was calculated for each year indicating the use of that topic in a particular year (i.e., based on sum of times, each keyword was mentioned each year, from 1975 to 2018). We then analysed how the three concepts have been linked together from 1975 to 2018 to explore the emergence of patterns in the linking of concepts over time.

## 3. Results

Searching the three core concepts separately yielded 405,636 publications. Publications were filtered to include only studies of humans, generating 263,751 publications from the three groups. This included studies related to the concepts of neuroplasticity (*n* = 6,498), stroke recovery (*n* = 79,060), and learning (*n* = 178,193).

**Figure 3:**
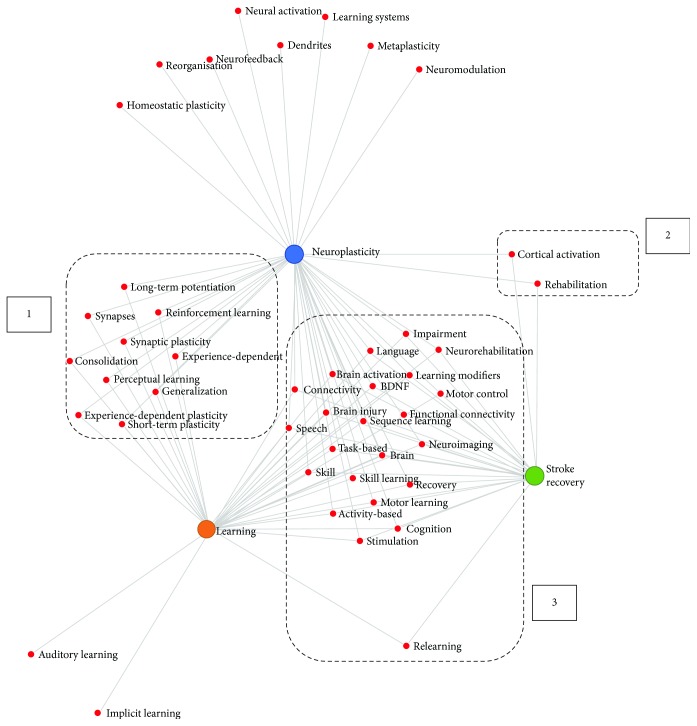
Generated concept map using the automatic text processing engine—showing 3 main *concepts* (nodes), strength of link between nodes (number of publications), identification of *common themes* being discussed based on the proposed concept link map (encircled areas 1, 2, and 3), and *topics* (words) that help to characterise the concept and/or the links between them.

Figure 3 illustrates the topical associations between the three main concepts generated from the automatic text processing engine following the concept map. The three main nodes in the generated concept map represent the focus areas: neuroplasticity, stroke recovery, and learning. Each line connected to the nodes represents topics discussed related to the respective research area. The strength of each line is an indication of the quantity of publications. The encircled components of the generated diagram are based on the proposed concept link map in the methodology. The numbers indicate the links between the concepts as follows:

1. Common themes being discussed in neuroplasticity and learning
2. Common themes being discussed in neuroplasticity and stroke recovery
3. Common themes being discussed in learning and stroke recovery with common themes in neuroplasticity

The common themes identified between the main concepts are listed in Table 2, together with an indication of the number of publications and normalised score (weights) for each theme.

The top 30 articles identified that intersect all three main concepts: neuroplasticity, learning, and stroke recovery, are listed in Table 3. These articles were selected according to their weighting and are ordered with the most recent at the top. It is noted that 15 articles are reviews and nine are controlled trials. The full text of these articles was downloaded and reviewed for the narrative review.

### 3.1. A Time-Based Analysis of the Terminology and the Evolution of Topics over Time

A post hoc analysis of the use of keywords (topics) for each concept and the evolution of how the topics link together over time revealed two outcomes: (1) overall topic distribution over time—this indicated how frequently a given topic was addressed in research studies each year thereby demonstrating the patterns over time; (2) the emergence of topics—this indicated when certain topics first appeared and how they evolved over time. Based on the patterns identified by these outcomes, we further examined the time-based topical associations to observe how the link (intersection) between the three concepts (neuroplasticity, learning, and stroke) had emerged over time. For demonstration purposes, we created three sets of publications based on the patterns detected by the time-based topic distribution. Three time periods emerged: (1) Early era (1975-1990); (2) Emerging era (1997-2003); and (3) Recent era (2012-2018). These time periods emerged primarily from the topic flow graph of neuroplasticity. Using the publications in these three groups, we analysed the evolution of the link between the three concepts. This process was automated by the proposed text mining approach.

**Figure 4:**
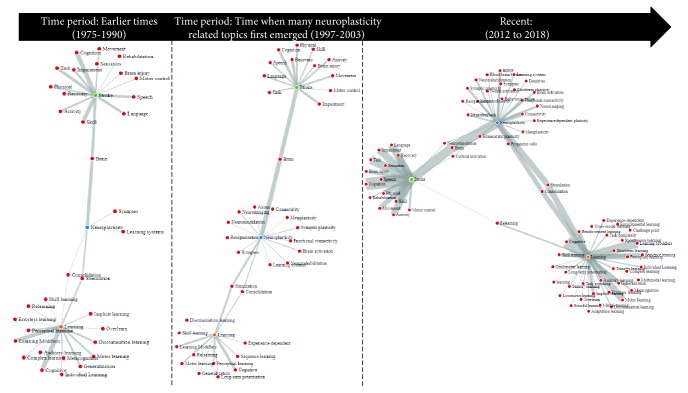
Generated comparison to demonstrate the evolution of topics over three selected time periods. The weight of the links is a representation of the quantity of publications.

Figure 4 highlights the outcomes of this analysis showing the associations of the concepts according to the aforementioned time periods. The Early era (1975-1990) was characterised by only a few topics in neuroplasticity. Prominent topics were “Stimulation,” “Consolidation,” and “Synapses.” The links between neuroplasticity, stroke, and learning are established. This was followed by the Emerging era (1997-2003), a time where many new topics (keywords) first appeared, particularly in relation to neuroplasticity, and more new directions of research were formed. The Recent period (2012-2018) revealed the latest research topics. Many new topics appeared in relation to all three concepts during this period. The link, Neuroplasticity-Stroke, was expanded with “Neurostimulation” and “Cortical activation” other than “Brain”; the link Neuroplasticity-Learning became stronger, with many more research studies; and the link Learning-Stroke emerged, linking all three concepts together.

## 4. Discussion

The aim of this review was to identify the literature that links neuroplasticity, stroke recovery, and learning in order to advance our understanding of and provide direction for a neuroscience-informed approach to stroke rehabilitation. The concept map generated by the text processing engine provides an efficient and rigorous approach to identify associations between different research areas as well as insights on important research themes and topics within a large pool of research publications. Moreover, the weighted link map provided a quantitative measure of the significance of the relationship between the themes; thus, the important topics could be identified. Finally, the intersection between all three concepts was defined and common topics identified. Time-linked patterns emerged from our analysis of the evolution of the link between the three concepts.

### 4.1. A Novel Methodology to Reveal the Presence and Absence of Topics and How They Are Linked

The methodology used to conduct this review is novel. Commonly, when commencing a literature review, a basic search term of interest will return a very large number of articles. Subsequently, more complex search terms are added until a manageable number of articles are returned. This often means there is little knowledge of the articles being excluded before the human reviewers' start to look at the final articles. The approach detailed here of conducting an extremely broad search of the literature databases and using natural language processing to understand what is present means the choice of articles to include and perhaps more importantly, knowing what is being discarded from review, has the advantage of being controlled and repeatable.

The intent of our approach was to identify key topics related to the core concepts in a systematic and comprehensive manner, thus scoping the currently available literature in the field. To achieve this, our approach employed a broad range of terms that represent the current literature and captured words that might have similar or overlapping meaning between studies and over time. The use of machine learning approaches involving text mining, word embedding, and natural language processing enhanced this feature of our review. However, there are two important considerations when conducting a literature search across different domains and across large spans of time. First, do the different domains use the same term to mean the same concept or are the same terms used to mean different things in their own domain? Second, has the meaning of a term changed over time or were concepts referred to by a different term in the past? Word embedding, which maps words to vectors of real numbers, can help with this, as it understands the context. The meaning of words and word relationships is derived from their use in the text rather than any dictionary definition. In line with this, it can describe what is in the current literature. It does not however attempt to define or evaluate the terminology used.

### 4.2. Themes and Topics Linking Neuroplasticity, Stroke Recovery, and Learning

The approach used allowed the existing literature to inform the themes and topics that link the three main concepts. In this way, it not only confirmed but also expanded the topics identified by domain experts. The topics identified that linked only two concepts were often quite specialised and limited. In comparison, 23 common themes/topics emerged from the intersection between all three concepts. This is reinforcing and provides direction to inform an integrated neuroscience and learning-based approach to rehabilitation.

Our major focus was on themes, or topics, at the intersection of all three concepts. *Cognition* was the major theme identified (see Table 3), highlighting the importance of this topic. The review of the top 30 articles identified that cognition was discussed both in the context of impairment of cognitive functions post-stroke (e.g., [35]) and in the context of cognitive and information processing perspectives involved in learning. The evolution of cognitive processing perspectives to a blended approach between neural science and social-cognitive psychological science was highlighted [44]. In addition, the importance of brain networks and systems that support cognition and its role in recovery and learning-based rehabilitation was evident. For example, a dissociation between disrupted memory modifications in the presence of normal consolidation was reported and may be related to differences in a lesioned brain structure linked with macrostructure network anatomy and microstructural white matter integrity [37]. Clearly, cognition is important, highlighting the need to recognise and assess cognitive profiles of stroke survivors, even those with reported mild neurological impairment. The issue of cognitive decline [60, 61] also needs to be considered.

As expected, *Brain* was also a topic that was represented in a large number of publications. As well as being a focus in its own right, it was often linked with terms such as brain function, brain damage, brain injury, brain plasticity, brain stimulation, brain imaging, brain activation, and brain networks. *Stimulation* was primarily referred to in the context of brain stimulation and adjunct therapeutic stimulation techniques, such as functional electrical stimulation (FES) [41]. This theme highlights the search for and possible role of adjunctive stimulation techniques to enhance neural plastic changes and stroke recovery. It highlights an area of research focus and proof of concept exploration of new therapies to try to manipulate plasticity and recovery.

Different types of learning were identified in the context of neuroplasticity and stroke recovery, representing a clear intersection of all three concepts (Link 3). These included *task-based learning* and *activity-based learning*. The common focus on learning in the context of tasks and/or activities (*n* = 3,970 publications) was identified using this approach. The topic of task-specific training, a term often used in clinical settings, was also aligned. These learning approaches are seen as potential enhancers of neural plasticity [49]. Task-based learning and activity-based learning map to concepts of learning-dependent plasticity. The role of learning that is task- and/or activity-based appears to have relevance in the context of stroke recovery and rehabilitation. For example, changes in central nervous system (CNS) structure and function may be modified by “activity,” together with motor learning principles [55]. In fact, both neuroscience and learning approaches that are integrated into rehabilitation included *task-based training* as a core element of therapy, consistent with recommendations [1, 9, 12, 23, 57].

Aligned with this focus on task- and activity-based learning is *skill* and *skill learning*, focusing on the outcomes of learning. Skill learning in the context of stroke recovery and neurorehabilitation links learning-dependent plasticity with restorative therapies. The goal of learning-dependent plasticity is often the learning of a skill, such as juggling and playing a musical instrument. In the context of stroke recovery, it may be learning a sensorimotor skill, such as learning to grasp a cup in a more normal manner following paresis. We have clear evidence from animal studies that training is a critical ingredient to this change [10, 62]. In human studies, evidence suggests that skill learning, but not strength training, induces cortical reorganization and cortical changes may only occur with learning of new skills and not just with repetitive use [9, 63]. For example, recent evidence highlights that motor skill learning of a repeated sequence altered cortical activation by inducing a more normal, contralateral pattern of brain activation, whereas increasing general arm use did not induce motor learning or alter brain activity [63].

A relatively large proportion of the publications (20.78%) were focused on *motor learning*, *movement*, and *motor control*. This finding highlights the current focus on movement outcomes, potentially at the expense of other functions or more complex outcomes. A relatively small proportion of articles focused on language and speech (9.2%). In comparison, focus on sensation (vision or touch) appeared to be missing as did more complex outcomes such as daily activities and or transfer to novel and/or complex activities. This likely reflects where the field currently is, i.e., in its infancy, in relation to applying knowledge that integrates neural plasticity with learning and valued stroke recovery outcomes. Nevertheless, the value of learning paradigms, in particular motor learning paradigms, is growing and a push to “infuse” motor learning research into neurorehabilitation practice is argued for in this literature [44]. An interesting observation was that the capacity for functional restitution after brain damage was different in sensory and motor systems [34]. The authors identified the role of adaptation and perceptual learning and their linkages with plasticity, as potentially important. Such findings further highlight the importance of systematic investigation across different functions.

Interestingly, *experience-dependent learning* was identified as a topic linking only neuroplasticity and learning (not the 3-way intersection) in our review (Link 1). Experience-dependent learning is closely aligned with experience-dependent plasticity [12]. Experience-dependent plasticity refers to the brain's capacity to change in response to environmental stimuli (and learning). It has been a major focus of preclinical studies and has culminated in the evidence of “enriched environments” to enhance recovery. Key features of this type of plasticity include exposure to environments that have multiple sensory attributes, social context etc. [12]. The potential for enriched environments to impact neural plastic changes and stroke recovery has been identified [8]; however, it did not emerge from the current review that represents the collective focus of the field. Given the existing link between experience and neural plasticity, the potential to connect this link more strongly with stroke recovery through targeted research is highlighted.

A few topics highlighted outcomes and/or mechanisms of change at a neurobiological level. Those topics that spanned underlying mechanisms or biomarkers included *connectivity*, *neuroimaging*, *BDNF*, *functional connectivity*, and *brain activation*. The neurobiological mechanisms underlying recovery in patients with varying severity of impairment and in the longer term, are incompletely understood. New technologies are emerging and have a role in providing new insights [64] and in helping to predict recovery and ability to benefit from interventions [36, 65]. For example, a predictive relationship was elucidated between the type of behavior, e.g., specific visual or distributed memory, and the brain lesion and network disruption [38]. This was possible using machine learning and multiple measures of the brain and behavior, i.e., resting functional connectivity (FC), lesion topography, and behavior in multiple domains (attention, visual memory, verbal memory, language, motor, and visual). A key role of distributed brain network disruption, beyond focal damage, was highlighted [38].

The process of and application of learning, including *sequence learning* to *relearning* and *neurorehabilitation*, were also identified as themes. Given the focus on learning and search terms used, it was interesting to note that the current literature often *did not* include topics that reflect a greater specificity in the nature of the learning, e.g., implicit and explicit learning. An exception was the identification of sequence learning as the approach to motor skill learning by Wadden et al. [36]. Again, this likely reflects the state of the science in the application these concepts to stroke rehabilitation. The issue of restitution of function, e.g., motor, versus adaptive motor learning strategies to compensate for motor impairments was identified but not resolved [39]. Nevertheless, we recommend this topic as an important avenue for future research on the basis that the process of learning is dynamic and could be disrupted following brain injury, and specific types of learning might be more beneficial following certain types of brain injury [23].

Of further interest is the fact that learning terms such as *generalization* and *transfer* (included in the MESH term for learning) did not emerge in any of the common themes. This is of potential concern given that outcomes associated with training and therapy need to be able to transfer to novel tasks and complex settings. The issue of sustainable and generalizable gains in motor skills and associated behaviors is highlighted in the rehabilitation literature [23, 57]. It is known that transfer to tasks that have not been directly trained in therapy is often very limited [57]. Transfer of gains in skills to personally-important real-life activities is rarely spontaneous and relatively rarely reported. Improvement in personally important, real-life activities is critical [23]. However, sensorimotor rehabilitation is historically focused on impairment reduction, with limited focus given to transfer of gains to real-life activities. Greater attention to outcomes that demonstrate different gradients of transfer and generalisation is recommended.

Neuroplasticity, learning, and transfer to novel tasks may be promoted by task complexity [12, 66, 67]. Different neural networks are implicated for learning of sensorimotor skills and transfer [68] and the value of metacognition strategies suggested [69]. The need for specific strategies to enhance transfer is supported by evidence from motor learning and neuroscience [68, 69]. Activity-dependent plasticity, defined as a form of neuroplasticity that arises from the use of cognitive functions and personal experience [67], would appear to be particularly relevant in this context. Interestingly, preliminary evidence suggests combined cognitive strategy and task-specific training improve transfer to untrained activities in subacute stroke [70].

Finally, *learning modifiers* was also identified as a topic. Factors that modify learning, its effectiveness, and impact at different times in the recovery trajectory are of interest. These factors ranged from factors such as BDNF [32] to adjunctive therapies, such as transcranial direct current stimulation [31] and robotics [42, 51]. One of the top 30 articles addressed the time course of skill reacquisition after stroke [46]. Other factors that might be modifiers of learning such as stress, concentration, perception, emotion, mood, and fatigue were not identified as topics despite being included as search terms.

### 4.3. The Evolution of Themes and Topics over Time

Further analysis was carried out to explore the evolution and associations of topics over time. Our objective was to observe how the topics in neuroplasticity, stroke recovery, and learning had evolved over time (1975-2018) using the collected sample of research studies from 1975 to 2018. Only a few topics were identified in the early time period (1975-1990). The link between neuroplasticity and stroke was established via research focused on “Brain,” while the link between neuroplasticity and learning was established via studies on “Stimulation” and “Consolidation.” In contrast, the Emerging era (1997-2003) showed the appearance of many more topics in neuroplasticity and the links have more weight indicating the availability of more research studies. The analysis of research in the Recent era (2012-2018) disclosed the emergence of many new topics. The link between neuroplasticity and stroke recovery was further expanded by studies on “cortical activation” and “neurostimulation.” It was also observed that the link between stroke recovery and learning was established in this time period, thus linking all three concepts together.

As this analysis was automated by the text mining approach described, further analysis and comparison using different time periods will allow disclosing other interesting patterns and insights regarding the associations among the three concepts. We present this time-based topic analysis as further contribution to the proposed approach as it enables researchers to mine useful time-based patterns from many publications without manual processing.

### 4.4. Recommendations for Future Research

Some recommendations for future research emerge from our review. The development of computational models of salient neural processes [40], including plasticity and learning systems of the brain in the context of stroke rehabilitation, is recommended. While focus to date has been primarily on motor function, we should not lose sight of the need to target other functions, such as language and sensation. Further, systematic investigation of outcomes across a profile of outcomes, including impairment and performance, activities, and participation is recommended [71] to achieve the valued outcomes articulated by people living with stroke [72]. We should also give greater attention to the processes of learning and how they map to different types of neural plastic changes, i.e. experience-dependent, learning-dependent, and activity-dependent plasticity. This is important as the different types of plasticity are aligned with specific goals, experiences, and learning conditions and may be more able to be enhanced at different times in the recovery trajectory. It is unlikely that one type of learning or principle of training, such as intensity, is likely to meet the skill and activity outcomes valued.

The development of future interventions should match neuroscience and learning principles to specific outcomes. In particular, the need to systematically target the intersect between neural plasticity and learning to achieve better generalisation of training effects and transfer to novel tasks in the context of stroke rehabilitation is critical. With further understanding, the potential to individualise therapy emerges. This may include the recognition of underlying capacities that support a particular type of learning, through genetic variations and strategies that influence modifiers of learning, such as BDNF. Finally, future research should be directed at discovering drivers of the different types of plasticity, as well as when they might best be applied at different times in the recovery trajectory.

## 5. Conclusions

In summary, the novel approach taken in this review allowed us to identify and characterise not only the topics that are currently being investigated in the literature but also those that are not or are only infrequently mentioned. Identification of the common intersecting themes linked with the core concepts proposed now provides a foundation of literature that may be synthesised to advance a neuroscience-informed approach to stroke rehabilitation. Further, such an approach helps to identify gaps in the field that may be important, as researched and recommended in related fields. For example, the topics of transfer and generalisation have been extensively researched in the field of learning, but did not emerge as an intersection with neural plasticity and stroke recovery. The review of the concepts of neural plasticity, learning, and stroke recovery and the common themes and topics that link them has provided direction for future research, important in the development of new neuroscience and learning-based therapeutic approaches. Finally, the potential also exists to develop theoretical frameworks by which new interventions may be conceptualised, incorporating knowledge of the intersection between contributing fields of research.

## Figures and Tables

**Table 1:** Domain knowledge from experts used for each of the three concept areas.

Concept 1: Neural Plasticity	Concept 2: Stroke Recovery	Concept 3: Learning	Concept 3: Learning *cont.*
Cells	Post-stroke	Experience-dependent	Activity-dependent
Synapses	Time	Experience	Adaptation
BDNF	Trajectory	Spontaneous	Transfer
Brain	Function	Implicit	Complex, complexity
Brain regions	Skill	Enriched environment	Metacognition
Neuroimaging	Impairment	Multisensory	Strategy
Learning systems	Movement	Multimodal	Problem solve
White matter	Sensation	Cross-modal	Generalise
Functional connectivity	Language	Long-term	Novel
Brain activation	Speech	Potentiation	Relearning
Reorganisation	Physical	Environment	Consolidation
Frontal	Cognition	Stimulation	Well learnt
Networks/systems	Mood	Performance	Overlearn
Brain network	Activity	Learning-dependent	Personal experience
Connection	Task	Skill learning	Environment
Behavior change	Work	Motor learning	Task complexity
Consolidation	Participation	Perceptual learning	Task switching
Experience-dependent plasticity		Sensory learning	Performance
Learning-dependent plasticity		Discrimination	Human
Activity-dependent plasticity		Generalisation	Individual
Glial cells		Reinforcement learning	Motivation
Microglia		Task-specific	Cognition/cognitive
Astrocytes		Sequence	Concentration
Gliosis		Errorful	Transmitters
Neuroimmunology		Errorless	Receptors
Blood brain barrier		Challenge point	Vision
Axons			Hearing
Dendrites			Perception
Circulation			Emotion
Neurogenesis			Mood
Progenitor cells			Fatigue
			Stress

**Table 2:** Common themes identified linking concepts of neuroplasticity, stroke recovery, and learning.

Topic	Normalized score	Publication count
Common themes between neuroplasticity and learning (Link 1)		
Synaptic plasticity	0.314	778
Consolidation	0.231	360
Long-term potentiation	0.145	340
Perceptual learning	0.145	280
Experience-dependent learning	0.059	150
Generalization	0.038	99
Experience-dependent plasticity	0.025	67
Short-term plasticity	0.022	58
Reinforcement learning	0.021	55
Common themes between neuroplasticity and stroke recovery (Link 2)		
Cortical activation	0.562	113
Rehabilitation	0.438	86
Common themes between neuroplasticity, stroke recovery, and learning (Link 3)		
Cognition	0.279	4032
Brain	0.141	3762
Stimulation	0.113	2830
Task-based learning	0.085	2136
Activity-based learning	0.073	1834
Motor learning	0.043	1090
Learning modifiers	0.041	1018
Skills	0.036	910
Movement	0.030	760
Impairment	0.029	732
Language	0.024	613
Connectivity	0.019	472
Speech	0.017	429
Neuroimaging	0.014	344
Neurorehabilitation	0.009	242
Motor control	0.008	203
BDNF	0.008	192
Skill learning	0.007	188
Functional connectivity	0.006	164
Brain injury	0.006	162
Brain activation	0.006	160
Sequence learning	0.004	107
Relearning	0.003	105

**Table 3:** Top 30 filtered articles that address common themes being discussed in learning and stroke recovery with common themes in neuroplasticity.

Author	Date	Title	Journal	Type
Charalambous et al. [30]	2018	The Feasibility of an Acute High-Intensity Exercise Bout to Promote Locomotor Learning after Stroke	*Topics in Stroke Rehabilitation*	Controlled trial
Fan et al. [31]	2017	Transcranial Direct Current Stimulation over Multiple Days Enhances Motor Performance of a Grip Task	*Annals of Physical and Rehabilitation Medicine*	Controlled trial
van der Vliet et al. [32]	2017	BDNF Val66Met but Not Transcranial Direct Current Stimulation Affects Motor Learning after Stroke	*Brain Stimulation*	Controlled trial
Pearson-Fuhrhop et al. [33]	2017	Genetic Variation in the Human Brain Dopamine System Influences Motor Learning and Its Modulation by L-Dopa	*PloS One*	Controlled trial
Horton et al. [34]	2017	Adaptation, Perceptual Learning, and Plasticity of Brain Functions	*Graefe's Archive for Clinical and Experimental Ophthalmology*	Review
Divya et al. [35]	2017	Post-Stroke Cognitive Impairment - A Cross-Sectional Comparison Study between Mild Cognitive Impairment of Vascular and Non-Vascular Etiology	*Journal of the Neurological Sciences*	Comparative study
Wadden et al. [36]	2017	Predicting Motor Sequence Learning in Individuals with Chronic Stroke	*Neurorehabilitation and Neural Repair*	Controlled trial
Censor et al. [37]	2016	Altered Human Memory Modification in the Presence of Normal Consolidation	*Cerebral Cortex*	Controlled trial
Siegel et al. [38]	2016	Disruptions of Network Connectivity Predict Impairment in Multiple Behavioral Domains after Stroke	*Proceedings of the National Academy of Sciences*	Clinical trial
Buma et al. [39]	2016	Brain Activation Is Related to Smoothness of Upper Limb Movements after Stroke	*Experimental Brain Research*	Clinical trial
Reinkensmeyer et al. [40]	2016	Computational Neurorehabilitation: Modeling Plasticity and Learning to Predict Recovery	*Journal of NeuroEngineering and Rehabilitation*	Review
Soekadar et al. [41]	2015	Brain-Machine Interfaces in Neurorehabilitation of Stroke	*Neurobiology of Disease*	Review
Kitago et al. [42]	2015	Robotic Therapy for Chronic Stroke: General Recovery of Impairment or Improved Task-Specific Skill?	*Journal of Neurophysiology*	Clinical trial
Lefebvre et al. [43]	2015	Neural Substrates Underlying Stimulation-Enhanced Motor Skill Learning after Stroke	*Brain: A Journal of Neurology*	Controlled trial
Winstein et al. [44]	2014	Infusing Motor Learning Research into Neurorehabilitation Practice: A Historical Perspective with Case Exemplar from the Accelerated Skill Acquisition Program	*Journal of Neurologic Physical Therapy: JNPT*	Case study
Mang et al. [45]	2013	Promoting Neuroplasticity for Motor Rehabilitation after Stroke: Considering the Effects of Aerobic Exercise and Genetic Variation on Brain-Derived Neurotrophic Factor	*Physical Therapy*	Review
Buma et al. [46]	2013	Understanding Upper Limb Recovery after Stroke	*Restorative Neurology and Neuroscience*	Review
Byl et al. [47]	2013	Effectiveness of Sensory and Motor Rehabilitation of the Upper Limb following the principles of Neuroplasticity: Patients Stable Poststroke	*Neurorehabilitation and Neural Repair*	Controlled trial
Bowden et al. [48]	2013	Promoting Neuroplasticity and Recovery after Stroke: Future Directions for Rehabilitation Clinical Trials	*Current Opinion in Neurology*	Review
Albert and Kesselring [49]	2012	Neurorehabilitation of Stroke	*Journal of Neurology*	Review
Arya et al. [50]	2011	Movement Therapy Induced Neural Reorganization and Motor Recovery in Stroke: A Review.	*Journal of Bodywork and Movement Therapies*	Review
Duret [51]	2010	[Contributions of Robotic Devices to Upper Limb Poststroke Rehabilitation]	*Revue Neurologique*	Review
Graham et al. [52]	2009	The Bobath Concept in Contemporary Clinical Practice	*Topics in Stroke Rehabilitation*	Review
Ween [53]	2008	Functional Imaging of Stroke Recovery: An Ecological Review from a Neural Network Perspective with an Emphasis on Motor Systems	*Journal of Neuroimaging*	Review
Ziemann and Siebner [54]	2008	Modifying Motor Learning through Gating and Homeostatic Metaplasticity	*Brain Stimulation*	Review
Daly and Ruff [55]	2007	Construction of Efficacious Gait and Upper Limb Functional Interventions Based on Brain Plasticity Evidence and Model-Based Measures for Stroke Patients	*The Scientific World Journal*	Discussion paper
Hlustík and Mayer [56]	2006	Paretic Hand in Stroke: From Motor Cortical Plasticity Research to Rehabilitation	*Cognitive and Behavioral Neurology*	Review
Krakauer [57]	2006	Motor Learning: Its Relevance to Stroke Recovery and Neurorehabilitation	*Current Opinion in Neurology*	Review
Forrester et al. [58]	2005	Exercise-Mediated Locomotor Recovery and Lower-Limb Neuroplasticity after Stroke	*Journal of Rehabilitation Research and Development*	Review
Winstein et al. [59]	1999	Motor Learning after Unilateral Brain Damage	*Neuropsychologia*	Controlled trial
